# Maximal muscular power: lessons from sprint cycling

**DOI:** 10.1186/s40798-021-00341-7

**Published:** 2021-07-15

**Authors:** Jamie Douglas, Angus Ross, James C. Martin

**Affiliations:** 1 High Performance Sport New Zealand (HPSNZ), Avantidrome, 15 Hanlin Road, Cambridge, 3283 New Zealand; 2 Cycling New Zealand, Cambridge, New Zealand; 3 Department of Nutrition and Integrative Physiology, University of Utah, Salt Lake City, UT USA

**Keywords:** Muscular power, Fatigue, Sprint cycling, Performance

## Abstract

Maximal muscular power production is of fundamental importance to human functional capacity and feats of performance. Here, we present a synthesis of literature pertaining to physiological systems that limit maximal muscular power during cyclic actions characteristic of locomotor behaviours, and how they adapt to training. Maximal, cyclic muscular power is known to be the main determinant of sprint cycling performance, and therefore we present this synthesis in the context of sprint cycling. Cyclical power is interactively constrained by force-velocity properties (i.e. maximum force and maximum shortening velocity), activation-relaxation kinetics and muscle coordination across the continuum of cycle frequencies, with the relative influence of each factor being frequency dependent. Muscle cross-sectional area and fibre composition appear to be the most prominent properties influencing maximal muscular power and the power-frequency relationship. Due to the role of muscle fibre composition in determining maximum shortening velocity and activation-relaxation kinetics, it remains unclear how improvable these properties are with training. Increases in maximal muscular power may therefore arise primarily from improvements in maximum force production and neuromuscular coordination via appropriate training. Because maximal efforts may need to be sustained for ~15-60 s within sprint cycling competition, the ability to attenuate fatigue-related power loss is also critical to performance. Within this context, the fatigued state is characterised by impairments in force-velocity properties and activation-relaxation kinetics. A suppression and leftward shift of the power-frequency relationship is subsequently observed. It is not clear if rates of power loss can be improved with training, even in the presence adaptations associated with fatigue-resistance. Increasing maximum power may be most efficacious for improving sustained power during brief maximal efforts, although the inclusion of sprint interval training likely remains beneficial. Therefore, evidence from sprint cycling indicates that brief maximal muscular power production under cyclical conditions can be readily improved via appropriate training, with direct implications for sprint cycling as well as other athletic and health-related pursuits.

## Key Points

- Maximal muscle power production under cyclical conditions is interactively constrained by force-velocity properties, activation-relaxation kinetics and muscle coordination across the continuum of possible movement frequencies.
- Fatigue alters the power-frequency relationship, with a higher degree of power loss at higher movement frequencies.
- Maximal muscular power production can be readily increased with appropriate strength and power training; it remains less clear if rates of power loss during brief maximal sustained efforts can be improved with training.

## Introduction

Feats of strength, speed and power have captivated humans for millennia and every 4 years, Olympic events which exhibit the (contemporary) limits of human performance are followed with intent by millions internationally. Such feats of human potential are fundamentally determined by muscular mechanical function, and especially, maximal muscular power. Indeed, outside the world of sport, maximal muscular power is often of life-or-death importance in predator-prey interaction and is important in health and disease. Track sprint cycling is an Olympic sport in which some of the most powerful athletes in the world generate remarkable speeds on a bicycle, with Olympic gold and fourth place often separated by only hundredths of a second. Previous investigators have reported that sprint cycling performance is largely determined by maximal muscular power production [1] and therefore serves as a useful model to investigate and advance the limits maximal muscular power by scientists and practitioners alike. Here, we present a synthesis of literature pertaining to physiological systems that limit maximal muscular power during cyclic actions characteristic of locomotor behaviours, and how they adapt to training, framed within the context of sprint cycling.

**Fig. 1:**
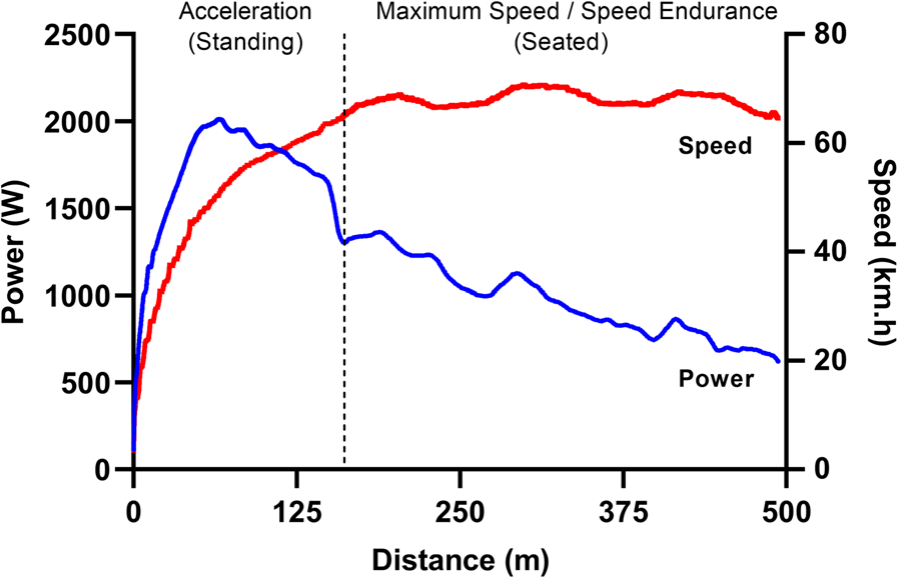
An example power profile and resulting speed from a highly trained male sprint cyclist during a maximal 500 m effort performed from a standing start (with an effort duration of 31.91 s). The dotted vertical line represents the transition from standing to seated cycling and is an approximate demarcation between acceleration and maximum speed/speed endurance phases

There are several sprint cycling events and currently three are contested at the Olympics (i.e. Match Sprint, Keirin and Team Sprint). Each event has its own nuanced technical, tactical and physiological demands, but there is substantial cross-over and multi-event Olympic champions are not uncommon. Cycling performance (e.g. velocity or time) is determined exclusively by the balance between propulsive power and resistance [1–8]. Power demand may be divided primarily into the power required to overcome aerodynamic drag, rolling resistance and drive train friction, and to bring about a change in potential or kinetic energy [1, 2, 6, 7]. The relative importance of these terms is dependent upon the instantaneous conditions and can change within a sprint cycling event [1]. During brief maximal accelerations from low speed, the change in kinetic energy will consume most of the power (Fig. 1) [9], and is related to mass and acceleration [10]. Because maximal accelerations are often initiated from a slow rolling or standing start in sprint cycling, power demand is directly proportional to the combined mass of the bicycle and rider [1]. At steady state speeds above ~40 km/h on flat terrain more than 90% of power is required to overcome air resistance, which is related to air density, frontal area, shape and velocity [10, 11]. Air (or aerodynamic) resistance (force) is proportional to the square of air speed, and power is related to the product of air speed squared and ground speed. With increasing speed, an exponentially larger increase in power is required to achieve a further increase in speed [1, 5, 6, 10]. Therefore, rider aerodynamic drag which is most commonly quantified as the drag area (C_D_A; a term combining the rider drag coefficient and frontal area) will account for most of the power demand at high speed [1], and reinforces the advantages of drafting where possible during sprint cycling competition [12, 13].

Power supply is determined by neuromuscular and metabolic capabilities [6]. Accordingly, due to the very high levels of muscular power required to generate high movement velocities, sprint cyclists tend to be more mesomorphic, stronger and more maximally powerful than other (i.e. endurance) cyclists [14–17]. The constraints inherent to human muscle contractile function and bioenergetics mean that power supply is limited in both rate and capacity from aerobic and anaerobic energy sources [18, 19], and so a negative exponential power-duration relationship is observed during maximal efforts [20]. In addition, both maximal and sustained (i.e. for a given event duration) power production are pedalling-rate dependent [4, 6]. As sprint cycling is performed on fixed-gear bicycles, the pedalling rate is non-constant and dependent upon the interaction of gear ratio and speed. The optimal power supply strategy (i.e. pedalling rate selection via gearing, and pacing) will therefore be dependent upon individual athlete characteristics, event distance, technical and tactical requirements [18, 21, 22]. Increasing maximal power for a given bicycle-rider mass will be most beneficial to improving the maximal rate of acceleration from a standing start, whilst increasing maximal and sustained power for a given C_D_A will be most beneficial to increasing maximum speed and speed endurance. Assuming technical/tactical competency and access to modern (i.e. aerodynamic) equipment [11], the sprint cyclist that exhibits the highest levels of maximum and sustained power relative to their body mass and C_D_A will be the fastest [23], and generally the most successful [24].

## Maximal Cycling Power

### Mechanical Basis of Maximal Cycling Power

Cycling is a motor task which involves the co-ordination the lower body prime movers operating in cyclic phases of shortening and lengthening to move the pedal (i.e. via the foot-pedal interface) in a circular trajectory at a given movement speed or pedalling rate, whilst applying the requisite force to the pedal necessary to achieve a given power output [25, 26]. Mechanical power is the product of force and velocity, or torque and angular velocity in the context of cycling [27–29]. Within fixed gear cycling, pedal forces directed normal to the crank (i.e. torque) will facilitate pedalling rate and power production; however, the increasing pedalling rate will then constrain maximal force and power via force-velocity and activation-relaxation effects (the ‘Physiological Basis of Maximal Cycling Power’ section). Power may be determined in several different ways, but it is generally reported as the average power produced by both legs over a half pedal cycle from top dead-centre to bottom dead-centre [30, 31], or a complete pedal cycle [1, 6, 32]. In research settings, specialised ergometers have been developed to measure torque, pedalling rate and power production [30–35], and in applied settings, power produced by sprint cyclists is typically measured via commercially available cranks instrumented with strain gauges (e.g. the SRM [Schoberer Rad Messtechnik Jülich, Germany] power metre) [1, 36, 37]. Maximum power values as high as 2400-2500 W and 25-26 W.kg^−1^ have been reported in elite male track sprint and BMX cyclists [1, 25, 38], and values of 20-23 W.kg^−1^ (e.g. ~1400-1600 W at a body mass of 70 kg) reported in elite female track sprint cyclists [39].

Males produce ~25% more maximal power than females due to a larger muscle mass [39–41], and possibly a higher relative area of muscle mass comprised of fast twitch fibres [42, 43], with muscle mass and fibre composition being strongly linked to maximal power production (the ‘Physiological Basis of Maximal Cycling Power’ section) [32, 44, 45]. Approximately ~80-85% of the power produced over a pedal cycle is generated during leg extension (i.e. the downstroke), whilst ~15-20% is produced during leg flexion (i.e. the upstroke) [26, 46]. This power is a product of joint-specific actions of the ankle, knee and hip, and by upper body actions which transfer power across the hip [46, 47]. Hip extension contributes the most to total power output during maximal cycling, followed by knee extension, knee flexion, and finally ankle plantarflexion [46, 47]. During standing cycling ~8-12% more maximum power may be produced versus seated cycling [48, 49] via a transfer of power across the hip from the upper body [6, 50]. Whilst it has been proposed that the lower body prime movers are not fully active (e.g. as ascertained from electromyographic [EMG] activity) during maximal cycling [51, 52], simulation data indicates that agonist musculature is operating at or near its maximal capacity [53]. Nonetheless, it should be acknowledged that the use of EMG data to assess muscle activation remains contentious [54], and a high degree of variability has been reported during maximal cycling [55]. Additionally, simulations of muscular behaviour rely on several assumptions which may not hold for all individuals or circumstances [53].

### Physiological Basis of Maximal Cycling Power

The maximal ‘fatigue-free’ power that can be produced during a cycling bout is determined by an interaction of intrinsic muscle properties, neural activation and constraints (e.g. movement velocity and time available to produce force) imposed by the task [56, 57]. Intrinsic properties governing muscle force production during cyclic contractions include the force-length and force-velocity (i.e. maximum force and maximum shortening velocity) relationships, activation-relaxation kinetics (i.e. the time required to activate and relax muscle following neural excitation) and history-dependent effects (i.e. force enhancement after active lengthening, and force depression after shortening) [56–60]. Given the direct relationships between crank length and muscle excursion amplitude, pedal velocity and muscle shortening velocity, and pedalling rate and excitation-relaxation kinetics, these variables interactively constrain power production during sprint cycling [57, 61]. Because the hip and knee extensors appear to actively lengthen immediately preceding shortening during maximal cycling [62, 63], there may be a history-dependent attenuation of the force-length effects on force production, especially at long muscle lengths [64]. This proposition is supported by the finding that pedal and joint-specific power production does not meaningfully change across a broad range (e.g. 145 to 195mm) of crank lengths [65, 66]. Force-velocity and activation-relaxation requirements placed on muscle are linearly coupled for a given crank length during cycling [58, 66, 67], and therefore maximal muscle power production during sprint cycling (i.e. for a given individual) is determined primarily by pedalling rate.

**Fig. 2:**
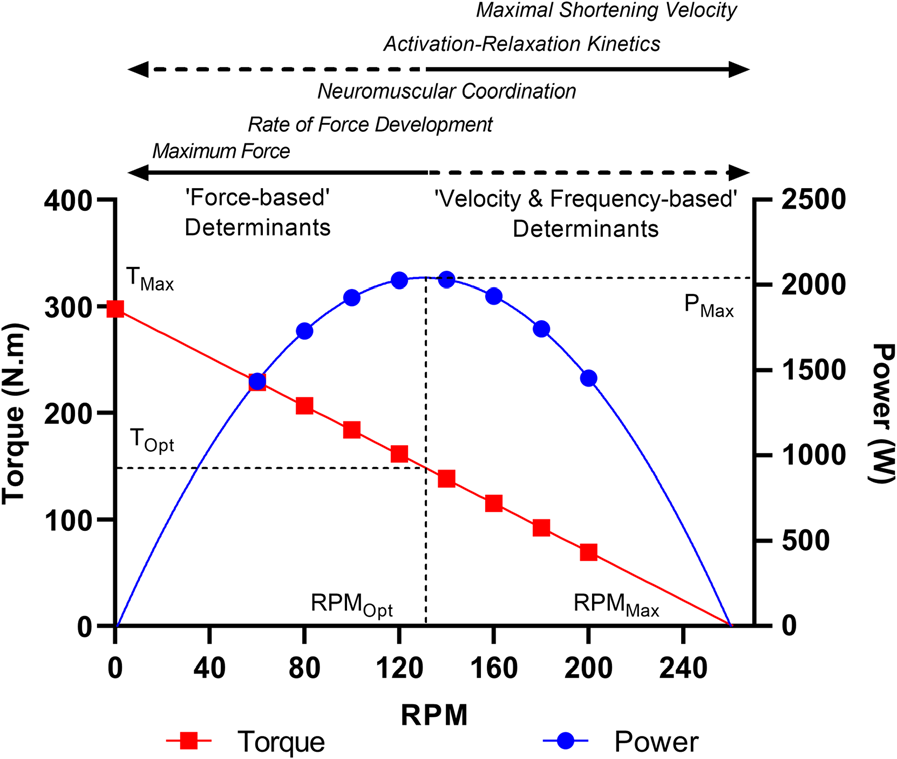
The torque- and power-pedalling rate relationship, parameters and determinants. The torque- and power-pedalling rate relationship is determined by an interaction of ‘force-based’ and ‘velocity & frequency-based’ factors. It should be noted that all determinants influence torque and power production at most pedalling rates experienced within sprint cycling; however, the relative importance of a given factor is pedalling rate dependent. Abbreviations: P_Max_, maximum power; RPM_Max_, maximum pedalling rate; RPM_Opt_, optimum pedalling rate (i.e. optimal frequency); T_Max_, maximum torque; T_Opt_, optimum torque

The relationships between torque, power and pedalling rate during cycling generally conform to the force-velocity and power-velocity relationships observed within isolated muscle [32, 34, 68], acknowledging that there is a linear rather than hyperbolic relationship between force and velocity during cycling [32, 38, 69–71]. Accordingly, there is a negative linear relationship between torque and pedalling rate, and a parabolic relationship between power and pedalling rate, with maximum power occurring at approximately half of the respective maximum torque and maximum pedalling rate values (Fig. 2) [23, 25, 32, 69]. The apex of the power-pedalling rate relationship typically occurs at an ‘optimal cadence’ (i.e. ‘optimal frequency’) of 120-130 rpm [6]. Higher optimal cadence values tend to be observed in conjunction with higher maximum power values [25, 72], which is unsurprising as both parameters are strongly linked to fast twitch (i.e. skeletal muscle fibres expressing a predominance of myosin heavy chain [MyHC] IIa and IIx isoforms) muscle fibre content [30, 45, 68]. An evenly mixed distribution of fast and slow twitch fibres across agonist musculature has been proposed to produce an optimal cadence of around 120 rpm in healthy non-power trained adults [30, 31, 45, 72]. Power trained athletes and individuals genetically endowed with a high proportion of fast twitch fibres may exhibit optimal cadences of 130 rpm and above [72–74].

Maximum muscle force and shortening velocity set the limits of the intrinsic muscle-force velocity relationship and power production at any given shortening velocity is determined by the interaction of these two parameters [28]. Maximum force will therefore directly influence the magnitude and rate of torque production at any given pedalling rate during cycling [28, 75]. The maximum force generated by a muscle fibre of any given MyHC isoform is directly proportional to its cross-sectional area [28, 76, 77]. Fast twitch fibre composition influences intrinsic force-velocity properties and muscle power production via a maximal unloaded shortening velocity that is 3-5 times faster than slow twitch fibres [28, 77–80]. Higher maximal shortening velocities in type II fibres are accompanied by sarcoplasmic reticulum Ca^2+^ handling kinetics and MyHC contractile machinery that allow faster rates of ATP hydrolysis, excitation-contraction coupling, and cross-bridge cycling [81–86]. Fast twitch fibres can therefore produce more force and power than slow twitch fibres at any given shortening velocity, with the effects being magnified with increasing shortening velocity [83]. In addition, for a given fibre type, fibre shortening velocity is proportional to its length, or number of sarcomeres in series [87]. All else being equal (e.g. muscle cross-sectional area), muscles that have longer fibre lengths have higher shortening maximal velocities, and therefore, a greater capacity to produce force and power at a given shortening velocity [28, 88, 89].

As pedalling rate increases during cycling, a greater proportion of the duty cycle becomes occupied by the processes of activation and relaxation, and therefore the role of activation-relaxation kinetics becomes increasingly critical to maximising positive work and minimising negative work [53, 56, 59, 61, 71, 90, 91]. For example, when cycling at 120 rpm, a full pedal revolution will take 500 ms, thus providing 250 ms for the shortening contraction to generate force. Even if rates of force development are high (e.g. due to high levels of neural drive and a brief electromechanical delay) [75], the minimum time required to activate and relax muscle will compromise the attainment of maximum force [6], and the total work that can be produced during a pedal cycle [57]. The minimum time required to deactivate or relax muscle may be 4-6 times longer than that required to activate muscle [53, 90, 92–94]. Accordingly, unrealised work resulting from time required to relax muscle is greater than that resulting from time to activate muscle [59]. Therefore, at most pedalling rates obtained within sprint cycling, the average force production over the pedal cycle will be substantially constrained by activation-relaxation kinetics [57], with relaxation kinetics being the more prominent limiting factor [59]. Ca^2+^ handling kinetics and cross-bridge cycling rates are the primary determinants of activation-relaxation kinetics [56, 90, 91, 95], and are primarily fibre-type dependent [83]. As noted, type II fibres are known to have faster Ca^2+^ handling and MyHC contractile machinery than type I fibres [81–86] allowing faster shortening velocities, and rates of force development and relaxation [68, 84, 86, 96]. In addition, series elastic component stiffness will influence the electromechanical delay and the speed at which muscle force can be transferred to the pedal following muscle contraction [75, 97, 98].

Whilst neural drive influences the rate and magnitude of force production at any given pedalling rate during sprint cycling [75], the ability to effectively coordinate the lower body synergists may limit the ability to produce power at pedalling rates above optimal cadence [99]. Maximal muscle activation does not appear to be influenced by pedalling rate, but an earlier EMG onset of the hip and knee extensors within the pedal cycle occurs with increasing pedalling rate [51, 52], which may reflect a coordinative attempt to account for the reduced timeframe available to produce force [56, 90]. Additional coordinative adjustments at high pedalling rates may include a preferential recruitment of fast twitch motor units [100], and an earlier deactivation of slow twitch motor units [101], to minimise the detrimental effects of slower activation-relaxation kinetics on power production. Nonetheless, disruptions in limb and muscle coordination consistently occur at or shortly after optimal cadence [102]. An increase in ineffective force and unrealised work at high pedalling rates indicates that coordinative adjustments cannot successfully overcome the limitations imposed by minimum timeframes required for activation and relaxation, and there is a limit to how well the limbs can be coordinated to effectively orient force at high movement frequencies [26, 52, 99, 102].

### Developing Maximal Cycling Power

Increasing maximal cycling power production is readily achievable through various training modalities that address underlying force-, velocity- and frequency-based neuromuscular properties [25, 103–107] (Table 1). It seems that force-based (e.g. maximum force and rate of force development) determinants of power production are more modifiable with training than velocity- and frequency-based (e.g. maximal shortening velocity, activation-relaxation kinetics and neuromuscular coordination) determinants [23, 25], and so, the power-pedalling rate relationship may be raised with training, but rightward shifts are less common. It is for this reason that elite sprint cyclists tend to exhibit relatively homogeneous velocity-based capabilities but heterogeneous force and power producing capabilities [23], highlighting the importance of strength training in the long-term development of maximal cycling power [25, 108]. Nonetheless, velocity- and frequency-based capabilities remain critical in determining the limits of the power-pedalling rate relationship [25], and training should address the range of force- and velocity-based capabilities to maximise improvements in sprint cycling power production [25, 108, 109].

**Table 1:** The effects of various training methods on the determinants of maximal muscular power, sustained power production during brief maximal efforts, and rates of recovery

Determinants	Maximum force	Rate of force development	Neuromuscular coordination	Activation-relaxation kinetics	Maximum shortening velocity	Fatigue resistance	Muscle oxidative capacity
**Relevant mechanisms**	- Muscle CSA - MyHC IIa/x area ratio - MyHC IIa/x composition (positive effect) - Neural drive - Muscle architecture	- Maximum force - Neural drive - MyHC IIa/x area ratio - MyHC IIa/x composition (positive effect) - MTU stiffness	- Magnitude of muscle activation -Timing of muscle activation and relaxation	- MyHC IIa/x composition (positive effect) -Sarcoplasmic reticulum structure and function	- MyHC IIa/x composition (positive effect) - Muscle architecture	- MyHC IIa/x composition (negative effect) -Anaerobic substrate availability and enzyme activity -Metabolite buffering capacity -Pain tolerance	- Muscle CSA (negative effect) - MyHC IIa/x composition (negative effect) - Mitochondrial and capillary density -Oxidative enzyme activity
**Training methods for maximal power**							
Maximum strength training	↑↑↑	↑↑	-	↓	↓	↑	↓ (?)
Explosive strength training	↑↑	↑↑↑	-	- (?)	- (?)	↑	-
Eccentric strength training	↑↑↑	↑↑↑ (?)	-	↑ (?)	↑ (?)	↓ (?)	↓ (?)
Isokinetic strength and power training	↑↑ (?)	↑↑↑ (?)	↑↑↑ (?)	-	-	↑↑	↑
Specific cycling strength and power training (track or ergometer)	↑↑	↑↑↑	↑↑↑	- (?)	- (?)	↑	↑
**Training methods for sustained power and rates of recovery**							
Sprint interval ‘Speed-Endurance’ training (track or ergometer)	-	↑	↑↑	-	-	↑↑	↑
Long interval training (ergometer or road)	↓↓	↓↓	-	↓↓	↓↓	↑↑	↑↑↑
Endurance training (ergometer or road)	↓↓	↓↓	-	↓↓	↓↓	↑	↑↑↑
Repeated sprint training in hypoxia	-	↓ (?)	-	↓ (?)	↓ (?)	↑↑	↑↑↑
Single legged interval training	-	↓ (?)	-	↓ (?)	↓ (?)	↑↑	↑↑↑

Abbreviations: *CSA* cross-sectional area, *MTU* muscle-tendon unit, *MyHC* myosin heavy chain isoform. Training effect key: ↑↑↑, highly positive; ↑↑, moderately positive; ↑, possibly positive effect; ↓↓↓, highly negative; ↓↓, moderately negative; ↓, possibly negative; -, neither positive nor negative; (?), effect uncertain

A higher capacity to produce maximum force does not guarantee high rates of force development or power production [110, 111], but due to the linear force-velocity relationship, an increase in maximal force indicates a greater capacity to produce force and power across a range of movement velocities [104, 109, 112, 113]. Therefore, it is unsurprising that maximum force and rate of force development exhibit strong associations with maximal cycling torque and power production in trained sprint cyclists [114, 115]. Increased maximum force is underpinned by muscle morphological (e.g. increased muscle cross-sectional area), architectural (e.g. increased pennation angle and decreased fascicle length) and neural (e.g. increased motor unit recruitment, rate coding and synchronisation) adaptations [28, 116, 117], which can be readily achieved through traditional strength training methods [112, 113, 118, 119]. Accordingly, traditional strength training remains a cornerstone of a sprint cyclists training regime [25].

Whilst novice (i.e. without a resistance training background) athletes can experience increases in strength and power in response to non-specific strength training stimuli [108, 112, 120], a foundation of maximum strength achieved through strength training is probably optimal in maximising long-term power development [108, 121]. Improvements in maximum force become increasingly difficult to achieve as strength levels increase and may translate less directly to improvements in high-velocity force production [108], indicating the need for greater specificity (e.g. movement pattern and velocity) and/or variation (e.g. via varied prescription or the introduction of novel methods) in training stimuli to achieve further increases in maximal power [112, 118, 121, 122]. For example, novel strategies such as velocity-based training [123, 124], eccentric training [125–128], isometric training [74] and electromyostimulation [129, 130] incorporated alongside traditional strength training may be effective in inducing further increases in maximal and high-velocity force production (e.g. via enhanced neural adaptations, preferential fast twitch fibre hypertrophy and increased muscle-tendon unit stiffness) in strength trained individuals. Upon the attainment of high levels of maximum strength, explosive strength training may become increasingly important to achieve further increases in maximal power [108, 110, 111, 121] via neural adaptations that increase the magnitude and rate of force development at high movement velocities [75, 108, 109, 131–134]. Excessive attention to heavy and slow strength training in the absence of explosive movements seems to be sub-optimal for power production [110], especially during cyclic tasks at high movement frequencies due to a slowing of relaxation kinetics [135, 136].

Whilst a foundation of traditional strength training will benefit maximal cycling power, there is a clear biomechanical discrepancy between the acyclic bilateral movements (e.g. squats and Olympic lifts) often implemented in practice and the cyclic unilateral demands of sprint cycling. Cycling-based force and power training likely remain critical to maximising the transfer of general neuromuscular strength and power to specific sprint cycling power production. Isokinetic cycling may be an especially effective means to maximise the transfer of general strength to cycling-specific force and power production at a given pedalling rate [105]; however, evidence supporting the utility of this modality is scarce. Alternatively, recent evidence indicates that cycling-specific isometric training can increase maximum cycling force and power production in elite sprint cyclists [74]. Resistive forces are readily modifiable within track cycling (e.g. via the manipulation of gear ratios, inertia, gravitational and aerodynamic forces), and so any given portion of the power-pedalling rate relationship may be addressed within specific training. Specific sprint cycling training utilising short duration efforts at high pedalling rates (e.g. ~160-210 rpm) seems to be effective in increasing power output at high pedalling rates in the absence of changes to MyHC composition and Ca^2+^ handling kinetics [104], potentially by improving neuromuscular coordination [51, 52, 99, 104]. Collectively, maximal and explosive strength training in conjunction with specific cycling power training can improve force-based properties and raise the torque- and power-pedalling rate relationship.

Improvements in velocity- and frequency-based capabilities may not be largely attainable independent of changes to MyHC composition [104, 137], and so are less responsive to training [25]. Shifting between fast twitch sub-fibre types (i.e. MyHC IIa ↔ IIx) can occur in response to training and detraining [138–142], but it is less clear if shifts between type I and type II fibres occur in humans [79, 143–145]. Sprint training does seem to induce a bidirectional shift (i.e. MyHC I → IIa ← IIx) with the slowest and fastest MyHC isoforms converging towards an intermediate isoform [146–148]. A period of detraining following resistance training may also induce an ‘overshoot’ of MyHC IIx composition above pre-training levels, largely at the expense of MyHC IIa fibres [141, 146], which has been associated with an increased maximal shortening velocity and high-velocity force production [149]. However, the detrimental effects of detraining on maximal force, power and fatigue-resistance [149–151], could possibly outweigh the positive effects of improved high-velocity contractile performance in trained sprint cyclists. Alternatively, some evidence indicates that eccentric training can increase MyHC IIx composition at the expense of MyHC I fibres [152], and indeed chronic eccentric training has been demonstrated to increase cycling power [153]. Irrespective of the possibility for changes in MyHC composition, eccentric training can induce a preferential hypertrophy of fast twitch fibres [125]. Whilst it is not clear if preferential fast twitch fibre hypertrophy influences maximum shortening velocity or activation-relaxation kinetics, an increase in the fast twitch to slow twitch area ratio may increase high-velocity force production [132, 136, 154, 155]. It is also plausible that eccentric training could increase maximal shortening velocity and power production at high pedalling rates via an increase in muscle fascicle length [89, 125], although this supposition has yet to be experimentally demonstrated.

Finally, sarcoplasmic reticulum volume density is a primary determinant of relaxation rates and high movement frequencies across species [156]. Therefore, very-high frequency training (e.g. on short-crank ergometers) has been used in practice to specifically induce quantitative and qualitative changes in sarcoplasmic reticulum excitation-contraction coupling machinery [157]. Nonetheless, at present, there is little evidence supporting the efficacy of this modality in modifying sarcoplasmic reticulum properties, although it may benefit power production at high pedalling rates (e.g. at or above optimal cadence) via improvements in neuromuscular coordination [104]. Whilst there may be little experimental evidence identifying changes in velocity- or frequency-based capabilities, it is important to note that small improvements have been reported over the career of elite sprint cyclists [25]. Such improvements may not dramatically improve maximal power production; however, it may be speculated that maintaining or subtly improving these qualities in the presence of extensive strength, sprint interval and endurance training (the ‘Developing fatigue resistance and muscle oxidative capacity’ section) may mitigate a shift towards a slower phenotype, and thus optimise long-term sprint cycling performance.

## Sustaining Maximal Power Production

### Fatigue-Related Impairments to Maximal Cycling Power

**Fig. 3:**
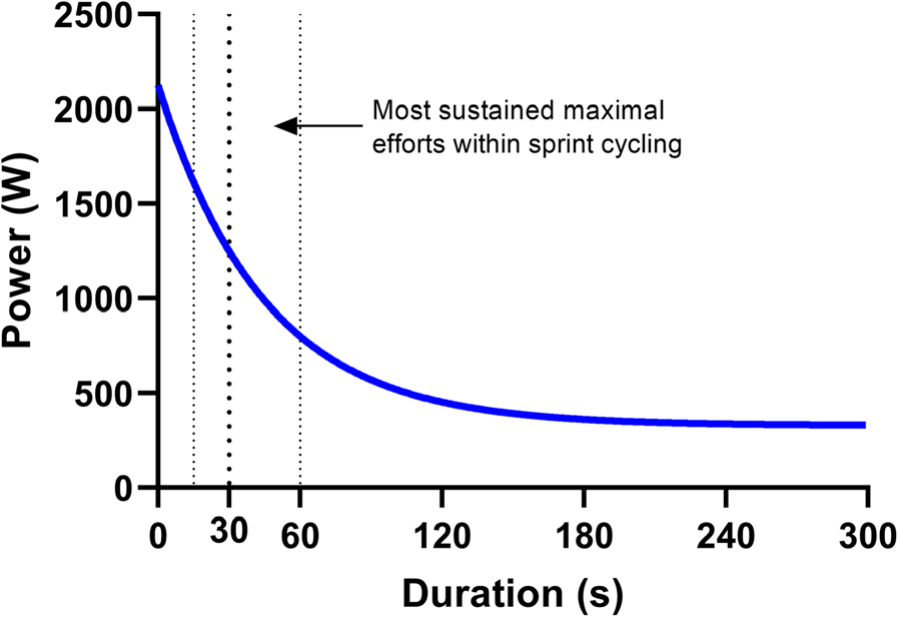
The power-duration relationship. Most sustained maximal efforts during sprint cycling last between ~15 and ~60 s, and so are characterised by a rapid exponential decay in power production. Extensive research into the mechanisms of sustained power production during brief maximal (i.e. ‘all-out’) efforts has utilised a 30 s (i.e. ‘Wingate’) exercise model

Maximal power is highly repeatable and fatigue resistant if there is a sufficient recovery duration between efforts [6, 158, 159]. However, the duration of most sprint cycling events requires sustained maximal efforts of ~15-60 s. Power production during human locomotion follows an exponentially decaying relationship with effort duration (Fig. 3) due to fatigue-related impairments in neuromuscular performance [20, 160–162]. During sustained maximal efforts power production declines from a maximal output at ~3 s to a near steady state after ~300 s [160, 162]. The power loss (i.e. also referred to as the ‘fatigue index’) during brief (i.e. ~25-30 s) maximal cycling efforts has been shown to be ~30-60%, or ~1-2% per s^−1^ [46, 68, 155, 163, 164], concomitant with a progressive downregulation of agonist muscle activation [165, 166]. A higher rate of power loss is evident with increasing pedalling rate [46, 68, 163, 164, 167], which likely reflects a leftward shift of the power-pedalling rate relationship with fatigue [168].

**Fig. 4:**
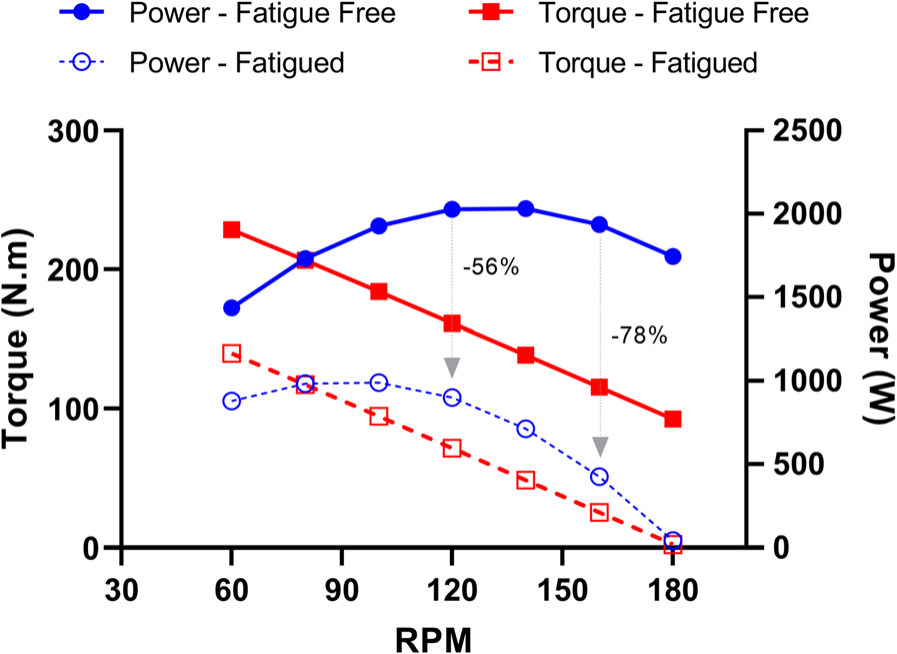
The torque- and power-pedalling rate relationship in the fatigue free and fatigued states (e.g. following 30 s maximal cycling). Because maximum force, maximum shortening velocity and relaxation rates are collectively impaired by fatigue; there is a reduction in maximum power (e.g. −56%) and a leftward-shift in the power-pedalling rate relationship. Accordingly, for a given ‘state of fatigue’, power production at high pedalling rates is compromised to a greater extent than at lower pedalling rates (e.g. −78% at 160 RPM vs. −56% at 120 RPM)

The high mechanical demands (i.e. necessitating the recruitment of fatigable fast twitch fibres) during sprint cycling places a substantial reliance on anaerobic energy production [169–171], and induces a progressive inhibition of contractile performance [20, 172, 173]. This is reflected in an altered power-pedalling rate relationship in the fatigued state [168, 174]. Fatigued fibres exhibit impairments in maximal force [175, 176], maximal shortening velocity [177, 178] and relaxation rate [179–181]. Maximum torque and maximum pedalling rate appear to be affected by fatigue at similar rates, collectively contributing to power loss [174], although unpublished data indicates that impairments in maximal force contributes most to power loss at low cycle frequencies whilst impairments in relaxation rates lead to the production of negative work and power loss at high frequencies (Link and Martin, In Review). A downwards leftwards shift (and possibly an increased curvature of the force-velocity relationship) is observed with fatigue (Fig. 4) [174, 175, 178]. Accordingly, impairments in both maximum power (e.g. ~45%) and optimal pedalling rate (e.g. ~31%) are seen following ~30 s of maximal cycling at the non-fatigued optimal pedalling rate [168]. The altered power-pedalling rate relationship may reflect the specific recruitment and fatigue of fast twitch fibres during maximal high-velocity tasks [68, 163, 182], and a subsequent reliance on fatigue-resistant slow twitch fibres to produce power [180], although this supposition remains to be experimentally corroborated.

Fatigue during brief maximal cycling seems to be largely peripheral rather than central in origin [143, 163, 183], although an interaction of mechanisms cannot be discounted [165, 184, 185]. Rates of ATP resynthesis per se may not limit performance during maximal tasks of ~60 s or less [20, 160, 161], rather contractile performance is downregulated when energy demands exceed rates of energy resynthesis [186]. Rates of high energy phosphate (i.e. ATP and creatine phosphate) depletion and inosine monophosphate (IMP) accumulation are indeed high during brief maximal cycling at high pedalling rates, especially in fast twitch fibres [187, 188], indicating a high degree of myocellular energetic stress, but an ensuing rigour state does not occur, and so excitation-contraction coupling mechanisms may be downregulated to avert a ‘metabolic catastrophe’ [186]. The higher rates of ATP hydrolysis and glycolysis in fast twitch fibres during maximal cycling likely results in a faster accumulation of metabolic by-products implicated in fatigue [83, 187, 189, 190].

The accumulation of the metabolites inorganic phosphate (P_i_), hydrogen (H^+^) and adenosine diphosphate (ADP) can interfere (i.e. directly or via a reduction of cytosolic pH) with glycolytic enzyme (e.g. phosphofructokinase) activity, myofilament sensitivity to Ca^2+^, cross-bridge kinetics (i.e. actomyosin binding number, force and cycling rate) and sarcoplasmic reticulum Ca^2+^ release and reuptake kinetics [95, 180, 184, 189–193]. These effects are especially pronounced within fast twitch fibre populations [194–196], and result in impaired force production, shortening velocity and rates of relaxation. Metabolite accumulation in the interstitial space also stimulates group III and IV chemo- and nociceptive muscle afferents [184], which may directly decrease motor output via inhibition of motor neuron recruitment and firing, and indirectly via a downregulation of central motor drive accompanied by sensations of discomfort or pain [162, 185, 191].

### The Importance of Aerobic Fitness Within Sprint Cycling

In many instances within sprint cycling maximum sustained power must be produced following a period of submaximal work, and often repeatedly following recovery periods ranging from minutes to hours. The magnitude of fatigue incurred from preceding submaximal work will be directly proportional to the intensity and duration of the bout, with rates of fatigue increasing with the relative reliance on anaerobic energy metabolism, as defined by the intensity relative to the maximum intensity that can be sustained by oxidative phosphorylation (i.e. critical power) [20, 160–162]. It appears that there is an approximately fixed amount of work (i.e. *W’*) that can be completed above the critical power before task failure occurs [19, 162]. *W’* seems to represent a combination of available biochemical stores and/or the maximal tolerable limit of metabolite-induced peripheral fatigue [197–200], with a depletion rate that is intensity-dependent [162]. Therefore, a higher critical power (e.g. via enhanced muscle oxidative capacity) will increase the range of submaximal intensities at which *W’* can be spared, thus mitigating fatigue-related impairments to subsequent maximal efforts.

The recovery of *W’*, which may reflect the resynthesis of high energy phosphate stores and clearance of metabolites, appears to be curvilinear with much of the repletion occurring within ~60 s [199]. Muscle oxidative capacity (i.e. capillary density, mitochondrial content and oxidative enzyme activity) is likely the most important factor influencing the recovery time course (i.e. encompassing metabolite removal, PCr resynthesis and restoration of cytosolic pH) following maximal fatiguing efforts [187, 199, 201, 202], although muscle carnosine content also predicts the rate of recovery of *W’* following hard exercise [199], presumably via an enhanced intramyocellular buffering capacity [203]. Muscle oxidative capacity tends to be lower in fast versus slow fibres [83, 204], and accordingly, the rate of recovery in these fibres is slower [143].

### Developing Fatigue Resistance and Muscle Oxidative Capacity

Increasing maximal power production is probably the most effective means to improve sustained power during brief maximal efforts via an overall increase of the power-duration relationship [107, 143, 205–208]. Indeed, reductions in rates of fatigue during ~30-45 s maximal cycling efforts seem to be difficult to achieve with training, even in the presence of physiological adaptations that would be expected to improve fatigue resistance [146, 147, 207, 209–211]. Nonetheless, addressing fatigue resistance through track- and ergometer-based sprint interval training (i.e. ‘speed-endurance’ training involving maximal sustained efforts with complete recovery) is probably still necessary to maximise sustained power production [212, 213]. It could be speculated that specifically addressing fatigue resistance following improvements in maximum power may be necessary to attenuate possible increased rates of substrate depletion and metabolite accumulation arising from enhanced metabolic and mechanical power output. An additional advantage of sprint interval training is an improved muscle oxidative capacity [213, 214], although specific aerobic training (e.g. endurance and other interval training variations) may still be necessary to ensure a sufficiently developed muscle oxidative capacity [213].

Improvements in fatigue resistance from sprint interval training could plausibly involve changes to enzyme activity, substrate stores and enhanced buffering of fatiguing metabolites [146], which may be reflected in an increase in markers of glycolytic flux (e.g. higher lactate production) for a given sprint cycling bout without any changes to local pH values [207, 211]. Sprint interval training seems to increase glycolytic enzyme activity which may increase maximal rates of glycolytic energy flux [107, 146, 209–211]. Whilst it may be possible to improve rates of anaerobic energy supply, it is not clear if supply is a rate limiting step to performance and rather energy supply rates seem to be largely demand-driven [20]. Attenuating the metabolite-induced downregulation of contractile performance through enhanced buffering may be the most relevant mechanism for improving fatigue-resistance with sprint interval training. The accumulation of metabolic by-products remains an unavoidable consequence of high rates of anaerobic energy supply and so it is not possible to entirely ameliorate the subsequent detrimental effects on contractile function. However, it seems pH regulation via H^+^ efflux and buffering is a reasonably modifiable avenue for performance enhancement [211]. Training-induced improvements in pH regulation may be related to adaptations to membrane transport systems for cytosolic H^+^ efflux (i.e. to mitochondria or extracellular buffers) [193, 215–218]. Monocarboxylate transporters (MCT1 and MCT4) account for most of the myocellular proton efflux during high intensity efforts [193], and are highly responsive to training and detraining [218], although less so in sprint-trained individuals [213].

It is possible that an improved tolerance to acidosis-related stimulation of type III-IV muscle afferents via repeated exposures could allow a better maintenance of motor drive in the presence substantial discomfort [191]. Whilst pedalling rate markedly influences rates of power loss within sprint cycling, little evidence is available identifying the optimal pedalling rate within sprint-interval training. It has been a common practice within elite sprint cycling to undertake training at very high pedalling rates (e.g. small gear track, ergometer or roller sprints) purportedly to elicit fatigue resistance specifically within the excitation-contraction coupling machinery. In contrast, the recent trend for larger competition gearing has resulted in faster competition times [219], and the practice of sprint interval training performed at low pedalling rates (e.g. large gear track or ergometer sessions) to elicit a form of ‘strength-endurance’ that could attenuate fatigue and power loss during sprint cycling. Based upon the principle of specificity, it seems intuitive to assume that the pedalling rate used within sprint interval training would mediate a particular adaptive response. However, this supposition has yet to be systematically corroborated. Indeed, a variety of training modalities, each intended to improve a specific limiting physiological mechanism may be the optimal approach [220].

Finally, improvements in muscle oxidative capacity can be readily achieved via traditional endurance and long interval training [221, 222]. However, excessive attention to this form of training may compromise the development of maximal strength and power [223]. Endurance training may directly downregulate rates of protein synthesis and inducing a shift towards more fatigue-resistant but slower MyHC (e.g. IIx → IIa → I) isoforms [79, 145, 146, 224], or compromise strength and power training via residual fatigue and/or substrate depletion [225]. Therefore, training to improve muscle oxidative capacity needs to be carefully dosed to mitigate potentially detrimental interference effects. Alternatively, novel training strategies may be implemented to more efficiently (i.e. achieving a given adaptive signal for a smaller dosage) elicit muscle oxidative adaptations. Emerging evidence indicates that relatively low volume short-term repeated sprint training (i.e. a protocol which elicits fast muscle fibre recruitment and high rates of oxidative flux) performed in hypoxia can induce non-haematological muscle adaptations associated with enhanced muscle glycolytic and oxidative capacities [226, 227]. Specifically, this form of training has been found to upregulate oxidative and glycolytic enzyme activity, muscle buffer content and mitochondrial and capillary density via an activation of the HIF-1α and HIF-2α signalling cascades [228–230], Similarly, single leg cycling is a training strategy which elicits an increased mechanical workload and local perfusion per leg during interval training versus double leg cycling, and subsequently has been shown to induce greater increases in muscle oxidative capacity in endurance trained cyclists [231]. Although it is not clear if the attenuated central stimulation from this method may compromise the maintenance of at least a minimum threshold of central (e.g. pulmonary diffusion capacity, cardiac output and oxygen carrying capacity) aerobic qualities. Further research is necessary to clarify the efficacy of hypoxic training and single leg cycling in improving muscle oxidative capacity in trained sprint cyclists.

## Conclusion

Maximal muscular power during cyclic contractions is limited by force-velocity and activation-relaxation characteristics, fatigue resistance and coordination amongst joints and muscles [26, 57]. At low cycle frequencies, maximal force and rate of force development may be most critical to power production [51], and as frequency increases to optimal frequency and beyond, maximal shortening velocity, activation-relaxation kinetics (especially relaxation) and muscle coordination likely play an increasingly prominent role [52]. Sprint cyclists operate at the edge of human potential with regards to maximal muscular power production, and those that exhibit the highest levels of maximum and sustained power relative to their body mass and aerodynamic drag will tend to be the fastest [1, 23]. Cycling power is a product of pedalling rate and the pedal force (directed normal to the crank) or torque (at the crank) generated from coordinated actions of the hip, knee and ankle extensors [25, 46]. Force-based determinants of maximum power are highly trainable [108], and traditional strength training combined with cycling-based strength and power training remains the foundation for long term power-development within sprint cycling. Velocity- and frequency-based determinants may not be highly modifiable with training independent of changes in MyHC composition [25]. Maximal efforts may need to be sustained for ~15-60s or longer during sprint cycling competition, but a rapid and progressive fatigue-related power loss is observed almost immediately after maximum power is attained [20]. The fatigued state is characterised by impairments in force-velocity properties and activation-relaxation kinetics, and so a suppression and leftward shift of the power-pedalling rate relationship occurs [168]. Increasing maximum power and raising the power-duration relationship is probably the most effective means of increasing sustained power during brief maximal tasks. It is unclear if rates of fatigue can be markedly improved with training even in the presence physiological adaptations associated with fatigue-resistance, although sprint interval training may still be necessary to optimise performance [213]. Traditional endurance training methods used to develop muscle oxidative capacity are known to interfere with maximal power development [145], and so the modality and dosage needs to be carefully considered. It may be postulated that a predominance of MyHC IIa fibres in particular and in combination with longer muscle fascicles (i.e. for a higher maximal shortening velocity) for a given muscle cross-sectional area may reflect an optimised phenotype for the simultaneous expression of maximal power, fatigue-resistance and muscle oxidative capacity [17, 89, 145], and therefore, sprint cycling performance. These insights can allow scientists and practitioners alike to better understand the mechanistic basis of maximal muscular power production, and subsequently, advance the limits of human potential within a range of athletic and health-related disciplines through the application of appropriate training strategies.

## Data Availability

Not applicable.
